# Vaccination with whole-cell killed or recombinant leishmanial protein and toll-like receptor agonists against *Leishmania tropica* in BALB/c mice

**DOI:** 10.1371/journal.pone.0204491

**Published:** 2018-09-24

**Authors:** Mosayeb Rostamian, Fariborz Bahrami, Hamid M. Niknam

**Affiliations:** 1 Nosocomial Infections Research Center, Kermanshah University of Medical Sciences, Kermanshah, Iran; 2 Immunology Department, Pasteur Institute of Iran, Tehran, Iran; Instituto Nacional de Salud Pública, MEXICO

## Abstract

One strategy to control leishmaniasis is vaccination with potent antigens alongside suitable adjuvants. The use of toll-like receptor (TLR) agonists as adjuvants is a promising approach in *Leishmania* vaccine research. *Leishmania* (*L*.) *tropica* is among the less-investigated *Leishmania* species and a causative agent of cutaneous and sometimes visceral leishmaniasis with no approved vaccine against it. In the present study, we assessed the adjuvant effects of a TLR4 agonist, monophosphoryl lipid A (MPL) and a TLR7/8 agonist, R848 beside two different types of *Leishmania* vaccine candidates; namely, whole-cell soluble *L*. *tropica* antigen (SLA) and recombinant *L*. *tropica* stress-inducible protein-1 (LtSTI1). BALB/c mice were vaccinated three times by the antigens (SLA or LtSTI1) with MPL or R848 and then were challenged by *L*. *tropica*. Delayed-type hypersensitivity (DTH), parasite load, disease progression and cytokines (IL-10 and IFN-γ) responses were assessed. In general compared to SLA, application of LtSTI1 resulted in higher DTH, higher IFN-γ response and lower lymph node parasite load. Also compared to R848, MPL as an adjuvant resulted in higher DTH and lower lymph node parasite load. Although, no outstanding ability for SLA and R848 in evoking immune responses of BALB/c mice against *L*. *tropica* infection could be observed, our data suggest that LtSTI1 and MPL have a better potential to control *L*. *tropica* infection and could be pursued for the development of effective vaccination strategies.

## Introduction

Leishmaniases are members of the neglected tropical diseases (NTDs), caused by intracellular protozoans of *Leishmania* genus which are transmitted via phlebotomine sand fly bites. There are three main forms of leishmaniases: visceral, cutaneous, and mucocutaneous. Approximately 350 million people live in areas with risk of leishmaniases in 98 countries. The disease affects 12 million people and its incidence is approximately two million cases per year [1]. *Leishmania tropica* is among the less-known *Leishmania* species which causes cutaneous leishmaniasis (CL) and rarely, visceral leishmaniasis (VL) in many endemic regions of the world [2]. Few studies have so far been dedicated to the pathology of *L*. *tropica* and the host immune responses against this parasite. Interestingly, the observed pathology of *L*. *tropica* in humans and experimental models is different from all other well-known *Leishmania* species, such as *L*. *major* [3]. These issues call for further studies on this source of a major public health problem, even more urgently.

Current available drugs for leishmaniasis are expensive and toxic. Moreover, there are reports that point to incidents of drug resistance by *Leishmania* species [4]. An alternative mean to control leishmaniases is vaccination with potent antigens in conjunction with suitable adjuvants.

Although several whole-cell, single purified, and recombinant *Leishmania* antigens have been used as vaccine candidates, so far there is no approved vaccine against leishmaniases [5]. Such antigens need suitable adjuvants to induce appropriate, long-lasting and protective immune responses [6]. Among the anti-leishmanial experimental vaccines, whole-cell killed vaccines have certain advantages such as high stability, high safety and low cost. Moreover, they can be manufactured in low-income endemic countries with relatively low technological skills [7, 8]. On the other hand, vaccines based on single purified or recombinant antigens also have many advantages, such as being well characterized and free from cellular products [9]. Since no approved effective vaccine for leishmaniases is available, research on both types of these prophylactics are ongoing.

Experimental animals are commonly-used models for exploring the immunological parameters of human leishmaniases. BALB/c mice are one of the most frequently studied animals for research on well-known *Leishmania* species, such as *L*. *major*. In contrast, few investigations have been done using experimental animal models to study *L*. *tropica* infection. The initial studies confronted with difficulties in setting up the infection in vivo [3]. However, it was revealed later that BALB/c mice could be a suitable model in this regard, due to chronic infection caused in these mice by different isolates of *L*. *tropica* [10–13].

To our best of knowledge, there is no report on vaccination against *L*. *tropica* in experimental animal models. Therefore, based on previous studies on *L*. *major*, here for the first time we tried to evaluate two kinds of *L*. *tropica* vaccine candidates (*i*.*e*. the whole-cell killed and a recombinant antigen) in an experimental animal model. For this purpose, *L*. *tropica* soluble *Leishmania* antigen (SLA) and recombinant LtSTI1 (a homologous protein of *L*. *major* stress-inducible protein-1, LmSTI1) were selected. LmSTI1 is a vaccine candidate which has shown protection against CL when used with suitable adjuvants [14–16]. *L*. *major* SLA combined with proper adjuvants have also been used as a whole cell killed vaccine and have exhibited protection potentials [17–20]. Currently, finding potential adjuvants which can direct and intensify specific immune responses and immunological memory by the innate immune response is a main objective to control complex pathogens such as *Leishmania* species.

Toll-like receptors (TLRs) agonists are molecules that interact with TLRs on the immune cells such as antigen-presenting cells. Such agonists are capable of activating the innate immune responses and can also trigger the function of the specific immune responses [21]. Thus, TLR agonists are interesting molecules to be used as adjuvants. There are several defined TLRs some of which are present in the cells endosomes (*i*.*e*. TLRs 3, 7, 8, and 9) and others are located on the cell membrane (*i*.*e*. TLRs 1, 2, 4, 5, and 6) [22]. The agonists for TLRs that facilitate the generation of T helper cell responses may be considered as T cell adjuvants [23]. This has been particularly important in the development of vaccines against pathogens that are controlled by cellular immune responses, such as *Leishmania* species [23].

TLRs 7 and 8 can naturally recognize viral single stranded RNA [24]. R848 (resiquimod) is a molecule, capable of activating both TLRs 7 and 8 [25] and has shown adjuvant capacity in many animal models [26]. Monophosphoryl lipid A (MPL) which can interact with TLR4 is a detoxified form of lipopolysaccharide (endotoxin) with strong adjuvanticity [27]. In our previous work, we tested the immune responses due to MPL and R848 adjuvants against *L*. *major* SLA in BALB/c mice [20].

In the present study, to further elucidate the effects of these two adjuvants, we used them alongside two different types of vaccine candidates (*i*.*e*. SLA and recombinant LtSTI1), against *L*. *tropica* infection.

## Materials and methods

### Parasite

The isolated *L*. *tropica* strain MHOM/AF/88/KK27 from Afghanistan was kindly donated by Dr. D. Sacks (Laboratory of Parasitic Diseases, National Institute of Health, Bethseda, Maryland, USA). The procedure of species confirmation and parasite culture were as mentioned in our previous reports [28, 29]. *L*. *tropica* parasites were cultured in Novy MacNeal Nicolle (NNN) media and then BALB/c mice were infected by the cultured parasites. *L*. *tropica* parasites were then retrieved from the mice’s lymph nodes for preservation of their virulence.

### Amplification, subcloning and nucleotide sequencing of *LtSti1*

*L*. *tropica* genomic DNA was extracted by a commercial QIAamp DNA Mini Kit (Qiagen, USA). *Leishmania* stress-inducible protein-1 (Sti 1) region is a conserved sequence in *Leishmania* genome which is called *LmSti1* in *L*. *major* and *LtSti1* in *L*. *tropica*. Based on well-known *LmSti1* sequence of *L*. *major* (NCBI GenBank accession number XM_001681088.1), the following primers were designed to amplify *LtSti1*: Forward, 5’-ACTAGGATCCGACGCAACTGAGC-3’; Reverse, 5’-GCTCGAATTCCTGACCAAAACGAATG-3’. Polymerase chain reaction (PCR) was performed using Exprime Taq enzyme (GeNet Bio, South Korea) which exhibits proofreading activity, under the following program: 94°C for 5 min, 30 cycles of 95°C—60 sec, 61°C—60 sec and 72°C—90 sec, and finally 72°C for 10 min. The PCR product was T/A cloned into pTZ57R/T using InsTAclone PCR cloning kit, (Thermoscientific, USA) according to the manufacturer’s instructions. pTZ57R/T plasmids containing *LtSti1* insert were extracted by TIANprep Mini Plasmid Kit (Tiangen, China) and sequenced by the plasmid’s universal primers (TAG, Denmark). Pairwise sequence alignment was performed using CLUSTALW program (http://www.ebi.ac.uk/clustalw/).

### Cloning, expression, and purification of LtSTI1

The *LtSti1* region was PCR amplified by the following forward and reverse primers, harboring *Nde*I and *Bam*HI restriction sites (underlined), respectively: Forward, 5’-GGACATATGGACGCAACTGAGCTAAAGAAC-3’; Reverse, 5’-TACGGATCCCTACTGACCAAAACGAATGAT-G-3’. The amplicon and pET-15b cloning/expression vector (Novagen) were digested by the corresponding restriction enzymes (*Nde*I and *Bam*HI) and the ligation reaction was set up at 1:1 molar ratio to insert the double-digested amplicon downstream of the vector-derived 6xHis-tag of pET15b in correct ORF using T4-ligase (Tiangen, China), according to the manufacturer’s instructions.

The recombinant pET-15b plasmid containing *LtSti1* construct was confirmed (by restriction analyses and DNA sequencing) and was transformed into *Escherichia coli* BL21 (DE3) for protein expression. The expression was induced by addition of 1M IPTG to *Escherichia coli* BL21 (DE3) culture harboring the confirmed plasmid and grown at 37°C (200 RPM) at OD600 ~0.5, and the growth was continued for 4 h after the induction. The expressed recombinant protein was confirmed by SDS-PAGE and Western blotting analyses using anti-HisG-HRP Antibody (Invitrogen, Carlsbad, USA), according to the manufacturer’s instructions.

The recombinant LtSTI1 protein which was expressed as insoluble inclusion bodies was purified by Ni-NTA agarose matrix (Qiagen, USA) with 8 M urea, according to the manufacturer’s instruction. Urea was removed using 10K-Amicon Ultra-15 columns (Millipore, Ireland), after 5 washing steps by LPS-free PBS buffer. The protein concentration was measured by Bradford assay [30]. Lipopolysaccharide (endotoxin) was removed from the protein solutions by Triton X-114 method as previously described [31]. The level of endotoxin was quantified by QCL-1000 Chromogenic Limulus amoebocyte lysate (LAL) test (BioWhittaker), according to the manufacturer’s instructions. All molecular assays and procedures were performed according to standard protocols [32].

### Antigens and adjuvants

*L*. *tropica* soluble *Leishmania* antigen (SLA) was prepared according to the procedure described for *L*. *major* SLA, previously [20]. Briefly, parasites were harvested at the stationary phase of the culture and washed 3 times with PBS buffer and then were subjected to freeze-thaw cycles (5 rounds) and centrifuged (16,000 ×g, 20 min, 4°C). The supernatant was collected as SLA and tested by sodium dodecyl sulfate polyacrylamide gel electrophoresis (SDS-PAGE). R848 and MPL adjuvants were purchased from Sigma-Aldrich (Germany) and Invivogen (France), respectively.

### Study animals

BALB/c mice (5–7 weeks old, female) were purchased from Pasteur Institute of Iran (Production Complex, Karaj, Iran). All experiments of the present study were approved by Ethics Committee of the Pasteur Institute of Iran (license number 95/0201/20704). The death of mice was not a likely outcome or a planned experimental endpoint due to our experimental manipulations, because according to the literature related to *L*. *tropica*, no death was presumed to occur during our experiments. Caring for and using the mice in this study were done according to “Iranian national ethical guidelines: How to work with laboratory animals”. Mice were maintained in the animal care facility under conventional conditions, kept in ventilated room in cages under 12 h of light and 12 h of darkness with unlimited access to water and food. Mice were monitored daily to assess animal health and well-being. Before removal of spleen and lymph nodes, the mice were euthanized by cervical dislocation.

### Immunization and infection

**Fig 1:**
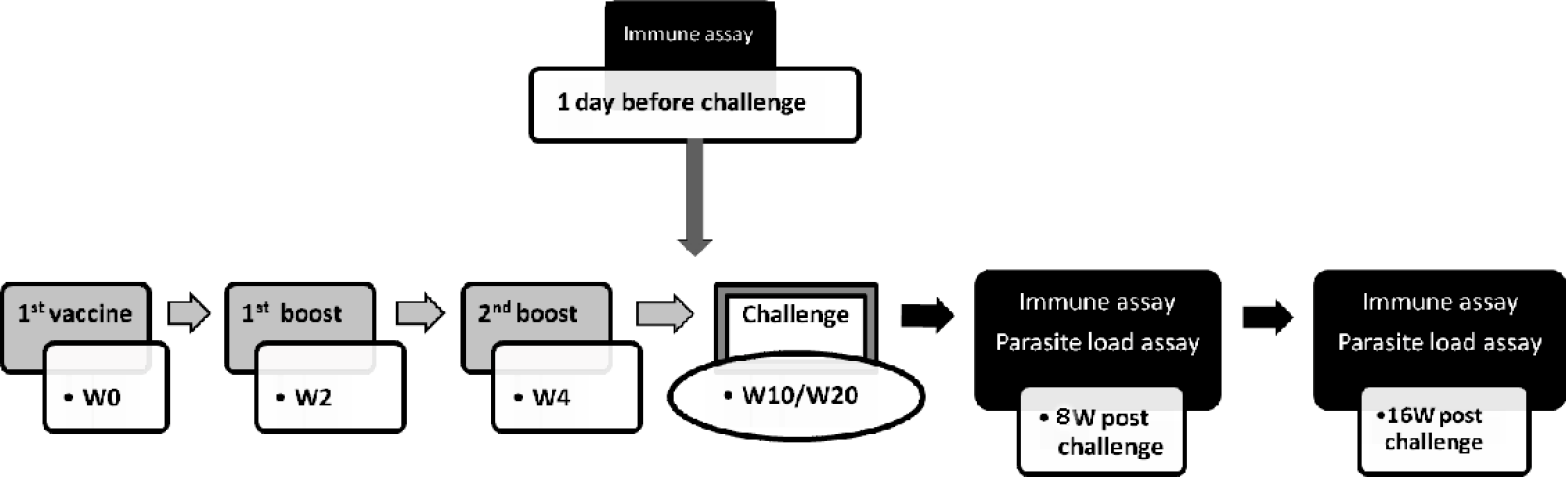
Schematic diagram of the vaccination, challenge and assays program. BALB/c mice were vaccinated 3 times with 2-week intervals and 6 weeks later (at week 10) were challenged by *L*. *tropica* promastigotes. In order to assay long-term immunity, 5 mice which had received LtSTI1 and the control groups were challenged, 16 weeks after the last vaccination (*i*.*e*. week 20). Three mice of each group were euthanized, 1 day before as well as 8 and 16 weeks after the challenge for the cytokines assays. W; week.

BALB/c mice were randomly divided into 7 groups (15 mice/group) as follow: LtSTI1 (indicated by “L”), LtSTI1+MPL (indicated by “LM”), LtSTI1+R848 (indicated by “LR”), SLA (indicated by “S”), SLA + MPL (indicated by “SM”), SLA + R848 (indicated by “SR”), and PBS. The vaccination, challenge and immune assay program were as shown in Fig 1.

TLR agonists (MPL and R848) were prepared according to the manufacturer’s instruction. In the adjuvanted groups, 20 μg of each adjuvant was applied to the vaccine preparation. Each mouse in the antigen-received groups obtained 25 or 10 μg of SLA or LtSTI1, respectively. Six weeks after the vaccination, each mouse was challenged with 2 ×10^6^ *L*. *tropica* stationary promastigotes. The percentage of morphologically metacyclic promastigotes in whole stationary phase promastigotes was ~25–35%, counted by Ficoll enrichment, as previously reported [33]. In order to assay long-term immunity of LtSTI1-received groups, 5 mice of these groups were challenged, 16 weeks after the last vaccination.

### Swelling measurements

The development of the swelling at injection site (right footpad) was monitored weekly using a dial-gauge caliper (Mitutoyo, Japan). The swelling values (in mm) were measured by subtraction of the thickness of the infected footpad from the thickness of an un-infected contralateral footpad.

### Assessment of delayed type hypersensitivity (DTH)

Twenty-four hours after the challenge, DTH was evaluated by measuring the thickness of the infected footpad, similar to measuring of the lesion, as mentioned above.

### Parasite load assay

Parasite load was measured by Real-Time PCR method, as previously mentioned [34, 35].

Briefly, DNA of the spleens and the draining lymph nodes were extracted using DNeasy Blood & Tissue Kit (Qiagen, USA). Equal dilutions of each sample (10 ng/μl) were made. Real-time hot-start PCR was performed in final volumes of 25 μl, using SYBR green master mix and the following primers that target the conserved region of the kinetoplast DNA (kDNA1 primers): Forward, 5’- GGGTAGGGGCGTTCTGC-3’; Reverse, 5’- TACACCAACCCCCAGTTTGC-3’. The thermocycling program was 95°C—4 min, 42 cycles of 95°C—10 sec and 60°C—35 sec, followed by a melting analysis. To quantify the parasites, a standard curve was generated using different dilutions of 10^8^ to 10^2^ copies of *L*. *tropica* genomic DNA and the parasite loads of the samples were determined by interpolation from the standard curve.

### Cytokines measurements

One day before the challenge, and also 8 and 16 weeks after *L*. *tropica* infection, draining lymph nodes of the mice (3 mice of each group) were harvested and cultured in presence of 10 μg/ml of antigen (LtSTI1 or SLA) or 2 μg/ml of Concanavalin A (as a positive control). Un-stimulated cells were used as a negative control. After 72 h, culture supernatants were collected, and IL-10 and IFN-γ levels were measured by DuoSet ELISA kits, according to the manufacturer’s instructions (R&D system, USA). These ELISA kits had a detection limit of 31.2–2000 pg/ml.

### Statistical analysis

To perform multiple comparisons of more than two groups, one-way analysis of variance (ANOVA) with Tukey’s post hoc test were used. The student’s t-test was used for comparison between two groups. A *p* value ≤ 0.05 was assumed to be significant. The mean value ± standard deviation (SD) or standard error of the mean (SEM) was applied to express the data.

## Results

### Characterization of *LtSti1* sequence

Sequence of *LtSti1* DNA was determined and submitted to NCBI GenBank (Accession No. KU744840). The alignment of *LtSti1* and *LmSti1* (GenBank accession No. XM_001681088.1) showed 98.2% homology at nucleotide level.

### Characterization of recombinant LtSTI1 protein

**Fig 2:**
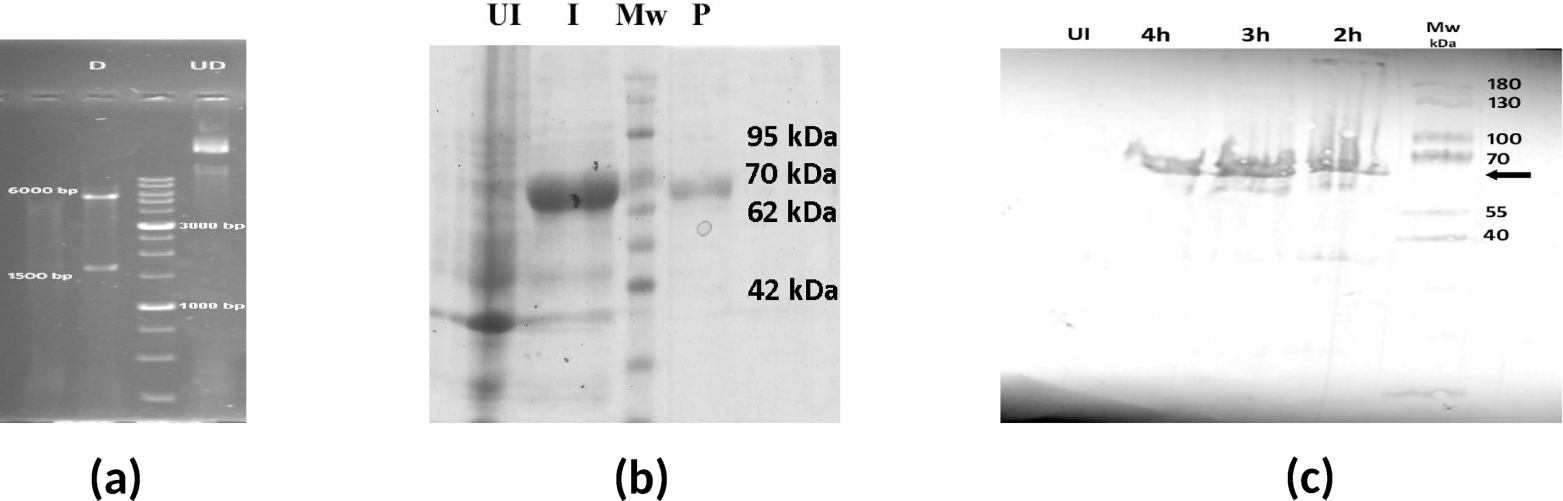
Cloning, expression, and purification of LtSTI1. (a) Cloning of *LtSti1* in pET15b vector was confirmed by double digestion of the final construct. Lane D, digested pET15b-*LtSti1* construct with *Nde*I and *Bam*HI restriction enzymes which showed two distinct bands of ~1641 bp and ~5708 bp, indicating *LtSti1* insert and pET15b vector, respectively; UD, undigested construct. (b) LtSTI1 protein expression and purification showed distinct ~64 kDa band on SDS-PAGE. Lane UI; crude extract of un-induced *Escherichia coli* (control), lane I; crude extract of IPTG-induced *Escherichia coli*, Lane P; purified protein, Lane Mw; molecular-weight marker. (c) Western blot analysis of LtSTI1 protein using anti-His monoclonal antibody. Crude extract of the induced *Escherichia coli* at 2, 3, and 4 h after IPTG induction are shown as 2h, 3h and 4h. The arrow indicates LtSTI1 protein. Mw, molecular-weight marker.

The final recombinant construct (pET15b-*LtSti1*) was verified by nucleotide sequencing and restriction digestion. Double digestion of recombinant pET15b-*LtSti1* construct by *Nde*I and *Bam*HI showed two distinct amplicons of ~1641 bp and ~5708 bp, indicating *LtSti1* insert and pET15b vector, respectively (Fig 2A). As expected, after induction by 1M IPTG, the ∼64 kDa band of LtSTI1 protein was visualized on 10% SDS-PAGE gel (Fig 2B). Also, a single ∼64 kDa band of the purified protein was observed on SDS-PAGE (Fig 2B). The expression of recombinant LtSTI1 was further verified by immunoblotting using an anti-His monoclonal antibody (Fig 2C). LPS removal by Triton-X114 method reduced the endotoxin level of recombinant LtSTI1 to lower than 3 endotoxin unit (EU), measured by LAL test (results not shown).

### DTH responses and parasite load results

**Fig 3:**
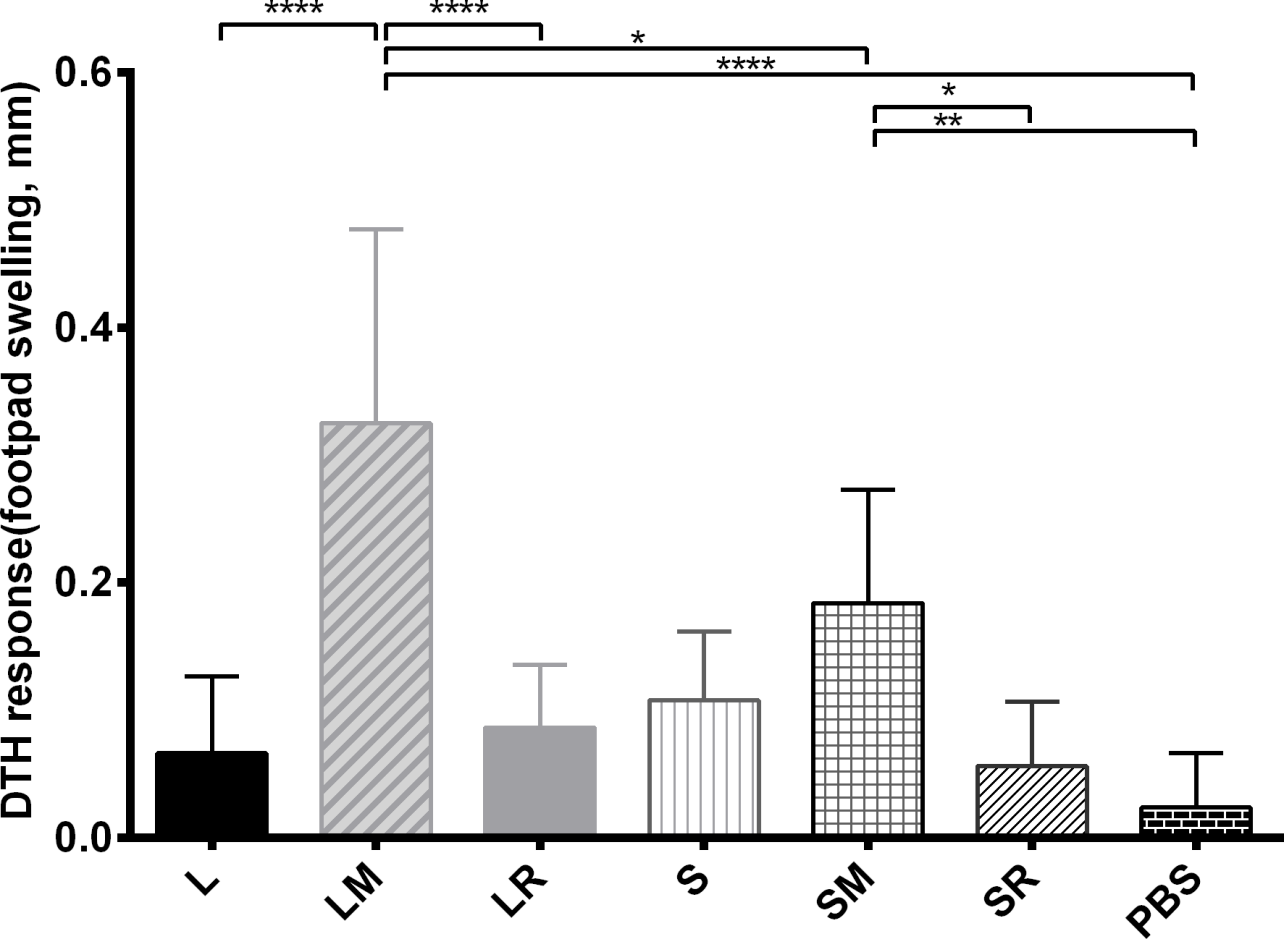
Delayed-type hypersensitivity responses. Six weeks after the last vaccination, the mice were challenged by *L*. *tropica* stationary promastigotes and 24 h after the challenge, their footpad swelling was recorded as DTH response. Each column shows mean +SD of the DTH value (7 mice per group). L, LtSTI1; LM, LtSTI1+MPL; LR, LtSTI1+R848; S, SLA; SM, SLA + MPL; SR, SLA + R848. Significant statistical differences are shown by asterisks (*, *p* ≤ 0.05; **, *p* ≤ 0.01; ***, *p* ≤ 0.001; ****, *p* ≤ 0.0001).

All the antigen-received groups caused a higher DTH response than PBS groups, although the difference was only statistically significant in some groups. Groups which received MPL plus antigen, caused a higher DTH response than the other groups. Moreover, LM group had a higher DTH response than groups that received SLA and MPL(SM group) as shown in Fig 3.

**Fig 4:**
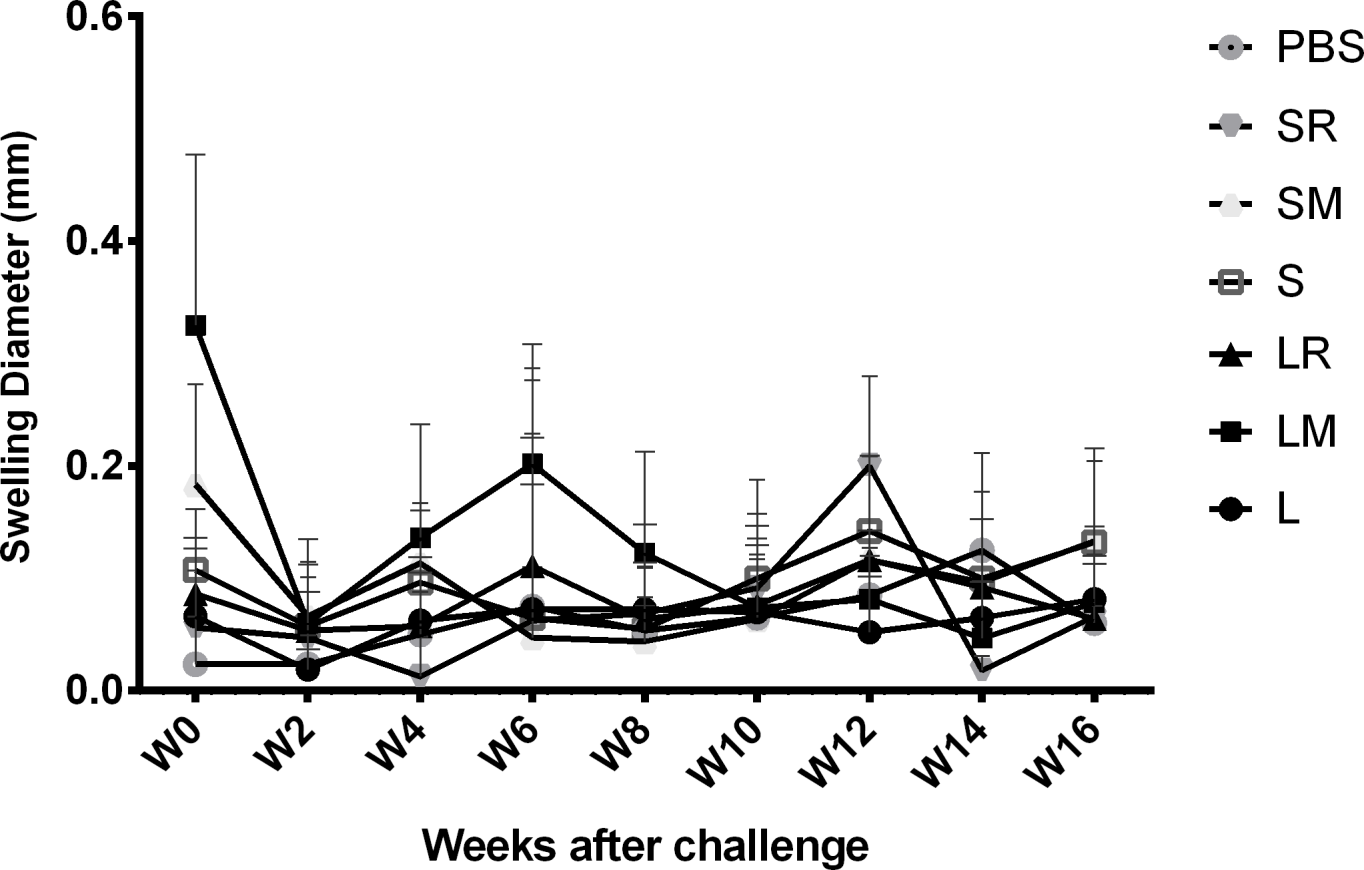
Swelling measurement after the challenge. Six weeks after the last vaccination, the mice were challenged by 2 ×10^6^ *L*. *tropica* stationary promastigotes. The swelling of the infected footpad was determined weekly. Each point shows mean of 7 mice per group L, LtSTI1; LM, LtSTI1+MPL; LR, LtSTI1+R848; S, SLA; SM, SLA + MPL; SR, SLA + R848.

Measuring the mice footpad thickness which was continued up to 16 weeks after the challenge, showed little or no significantly different swelling in our experimental groups (Fig 4).

**Fig 5:**
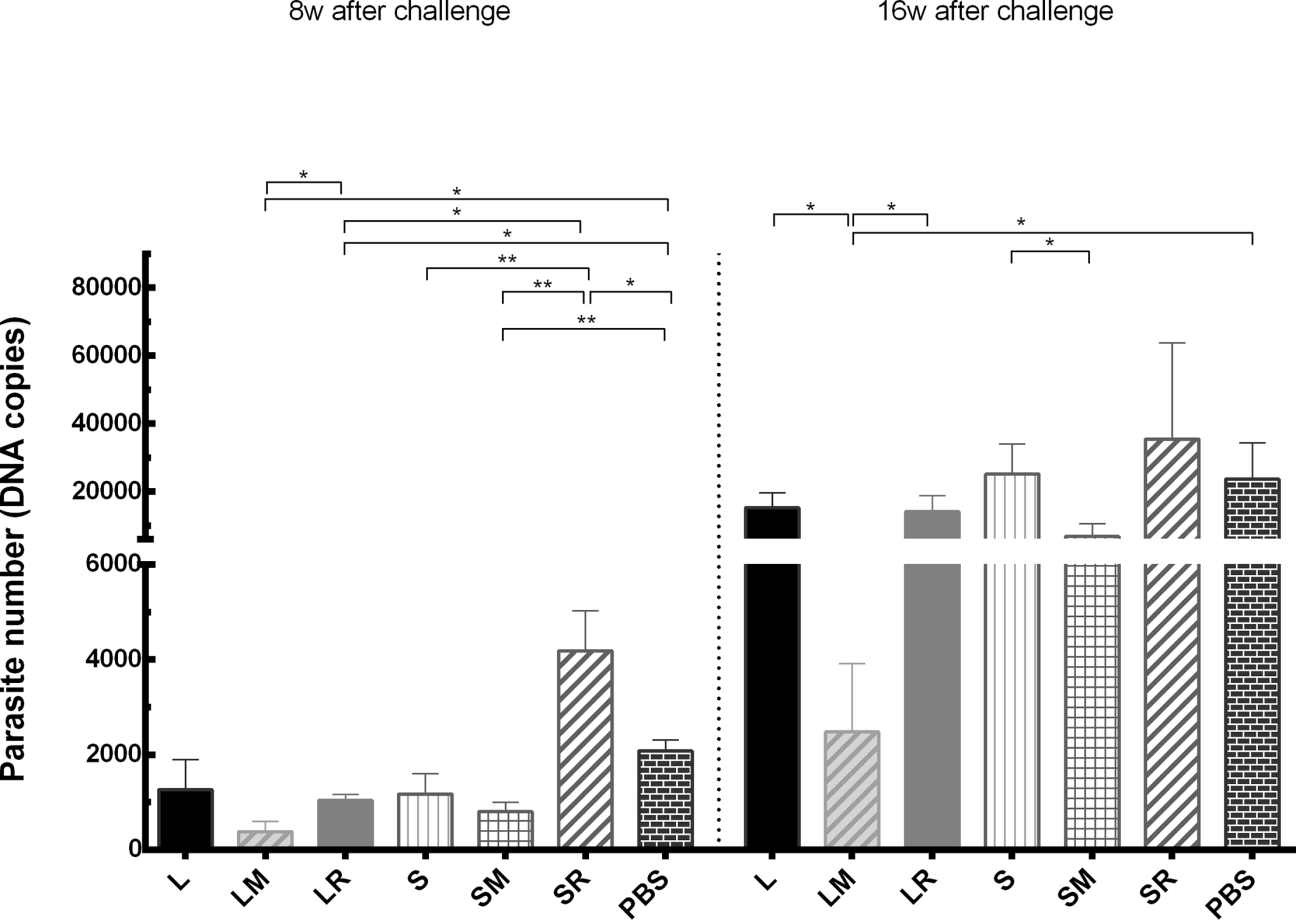
Parasite load of lymph nodes. Six weeks after the last vaccination, the mice were infected in the footpad by *L*. *tropica* promastigotes. At week 8 and week 16 after the challenge, the draining lymph nodes were removed and assayed for parasite load by Real-Time PCR. Significant differences are shown by “*” (*, *p* ≤ 0.05; **, *p* ≤ 0.01). Each bar shows mean + SEM of the parasite load (3 mice per group). L, LtSTI1; LM, LtSTI1+MPL; LR, LtSTI1+R848; S, SLA; SM, SLA + MPL; SR, SLA + R848.

No parasite was seen in any spleen samples of our experimental groups. Meanwhile, the results of the lymph nodes parasite load (Fig 5) showed an almost similar pattern in both assays, performed at 8 weeks and 16 weeks after the challenge; however, the parasite numbers were much higher at week 16, compared to week 8. Although all LtSTI1-received groups showed a lower parasite load than the control, the group which received MPL (LM group) had a lower parasite load than the others. In SLA-received groups, the lowest parasite load was seen in SM group. In SR group, the parasite load was higher than the control. Comparison of groups which received LtSTI1 (with or without the adjuvant) and SLA (with or without the adjuvant), showed that LtSTI1 was more potent in parasite reduction than SLA.

### Immune responses

**Fig 6:**
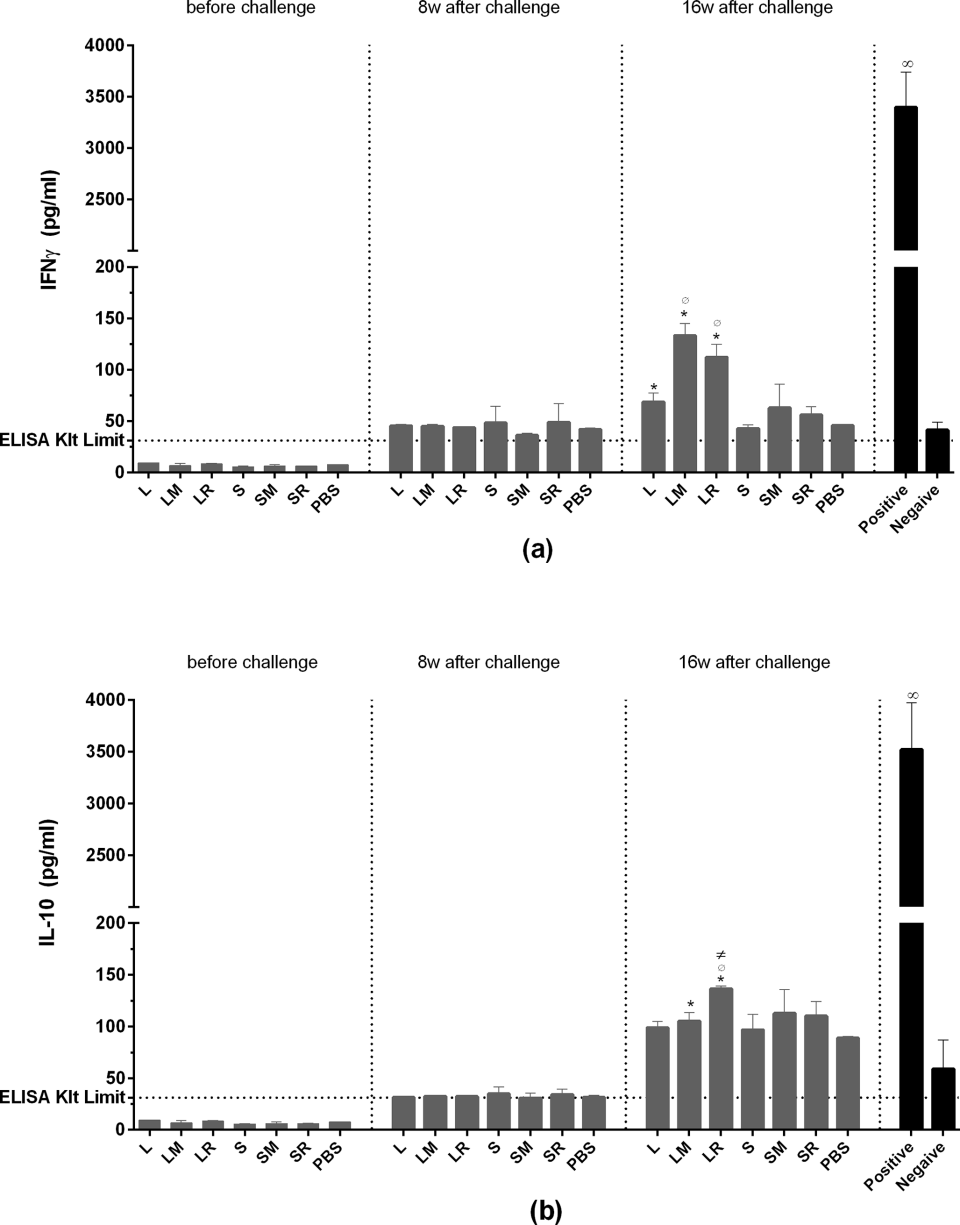
Cytokines responses. Before the challenge as well as 8 and 16 weeks after the challenge, the draining lymph nodes were removed and assayed for the cytokines levels. IFN-γ (a) and IL-10 (b) were assayed using ELISA kits. Before the challenge, the cytokines levels were lower than the ELISA kit limitation (shown by the horizontal dotted line). At week 8 after the challenge, the cytokines levels were not higher than the negative control (un-stimulated lymphocytes). At week 16 after the challenge, groups vaccinated by recombinant protein showed a cytokine level higher than PBS group (shown by “*”). Also at week 16, groups which received adjuvant plus LtSTI1 showed a higher cytokine response than LtSTI1 alone group (shown by “Ø”). The IL-10 level of group LR was slightly higher than LM group (shown by “≠”). The cytokine level of the positive control group was always higher than other groups (shown by “∞”). Each bar shows mean + SD of cytokine response (3 mice per group). *, *p* ≤ 0.05; Ø, *p* ≤ 0.05; ≠, *p* ≤ 0.05, ∞, *p* ≤ 0.05. L, LtSTI1; LM, LtSTI1+MPL; LR, LtSTI1+R848; S, SLA; SM, SLA + MPL; SR, SLA + R848.

Using ELISA, the concentrations of IFN-γ and IL-10 secreted from the cultured cells were assayed. None of our experimental groups showed a remarkable level of cytokine before and 8 weeks after the challenge. Before the challenge, the cytokines levels were very low and even lower than the detection limit of the ELISA kits (31.2 pg/ml). Although at week 8 after the challenge, the cytokine levels were higher than the detection limit, they were not higher than the quantities, corresponding to the negative control (un-stimulated lymphocytes). At week 16 after the challenge, groups vaccinated by the recombinant protein showed a cytokine level higher than PBS group. Higher cytokine responses were seen in groups which had received LtSTI1, compared to groups that had received SLA. Groups which received adjuvant plus LtSTI1 showed higher cytokine responses than groups with LtSTI1 alone. There was no significant difference between IFN-γ level of LM and LR groups; however, the IL-10 level of LR group was slightly higher than LM group (Fig 6).

### Long-term immunity assay

**Fig 7:**
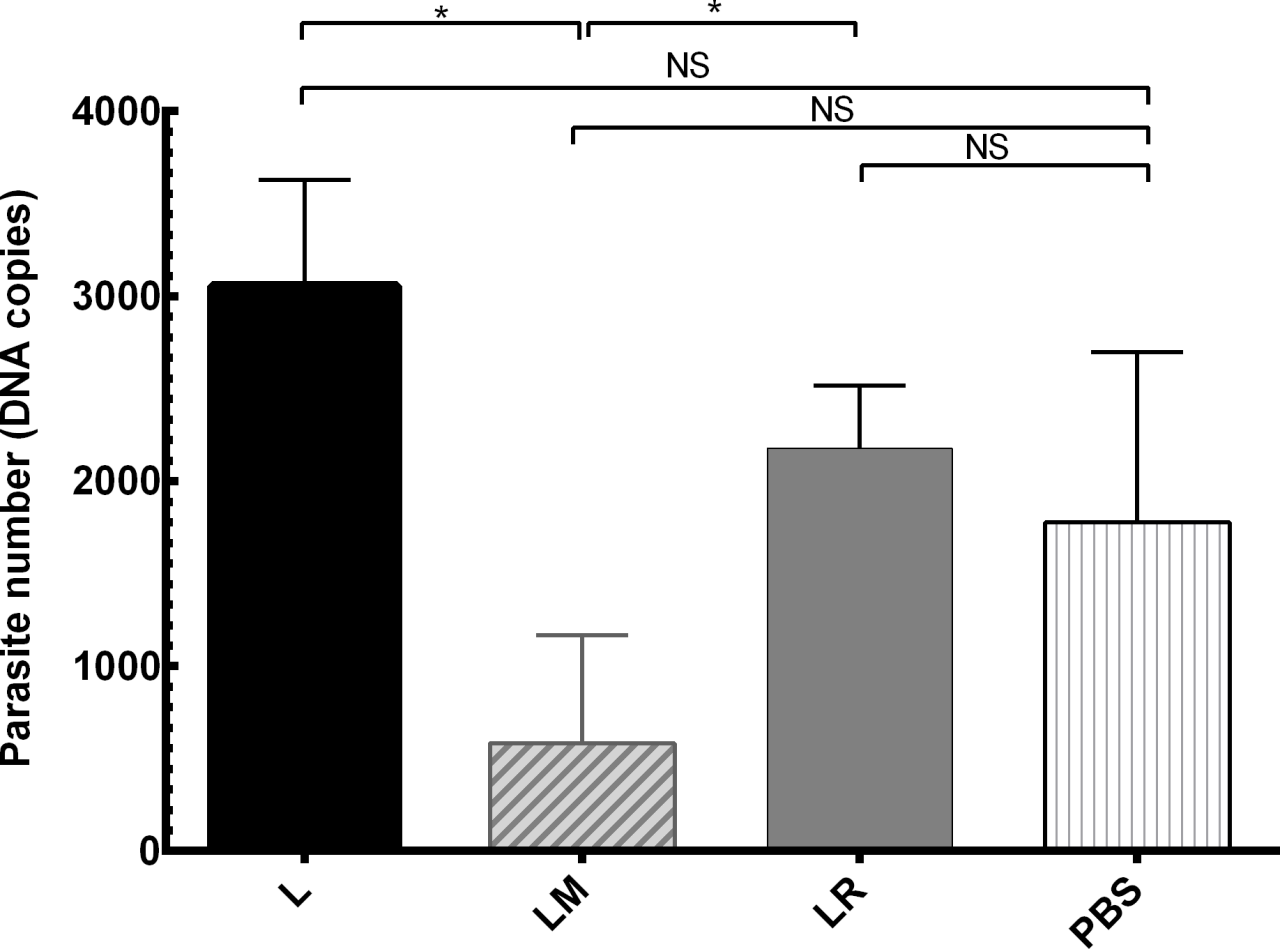
Parasite load of the lymph nodes in long-term immunity assay. Sixteen weeks after the last vaccination, LtSTI1-received and the control mice were infected in the footpad by *L*. *tropica* promastigotes. Six weeks later, the draining lymph nodes were removed and assayed for parasite load by Real-Time PCR. Significant differences are shown by “*” (*, *p* ≤ 0.05). NS, Not significant. Each bar shows mean + SEM of the parasite load (3 mice per group). L, LtSTI1; LM, LtSTI1+MPL; LR, LtSTI1+R848.

Sixteen weeks after the last vaccination, 5 mice of LtSTI1-received and the control group were challenged by *L*. *tropica*. Six weeks later, parasite load assays were performed by Real-Time PCR. The result showed almost the same pattern as the short-term immunity in which the parasite was absent in the spleen samples and MPL in contrast to R848 had decreased the parasite load in the lymph nodes, although none of our vaccinated groups showed lower parasite load than PBS group (Fig 7).

The cytokine levels of all groups in the long-term assay were as low as the negative control group (un-stimulated lymphocytes; data not shown).

## Discussion

In the present study for the first time, a homologous protein of LmSTI1 was identified in *L*. *tropica* and was produced recombinantly. Sequence analyses showed a high homology between *LmSti1* and *LtSti1*, indicating that this region is conserved between the two *Leishmania* species. The LtSTI1 protein was successfully produced in an *Escherichia coli* expression system and was applied as single protein vaccine candidate.

DTH is a T cell–dependent in vivo response. DTH is formed due to an inflammatory reaction which reaches its peak at 24 to 48 hours after the antigenic challenge [36]. The DTH responses show that none of our antigens were strong enough to cause a high DTH response alone. MPL, in contrast to R848, made both antigens (LtSTI1 or SLA) more capable of evoking immune responses. Similar to these results, we have previously shown that MPL is a better adjuvant in DTH evoking than R848 when is applied with *L*. *major* SLA [20]. Moreover, our DTH data here show that MPL worked better with recombinant LtSTI1 in comparison to SLA, suggesting that LtSTI1 may be a better vaccine candidate. There is no report on DTH response of vaccinated mice by LtSTI1 or *L*. *tropica* SLA; however, there are some reports indicating high DTH responses of vaccinated mice by *L*. *major* SLA or LmSTI1 plus adjuvants [16, 17].

No lesion or remarkable swelling was seen in our experimental groups. However, it should be considered that mild swelling of the footpad may not be detectable with routine methods such as dial-gauge caliper. Furthermore, in our other works using the same parasite species, we only were able to detect very low footpad swelling [37]. This indicates that *L*. *tropica* KK27 does not produce robust lesions in BALB/c mice. These results are consistent with previous studies which have clearly indicated that CL strains of *L*. *tropica* (such as KK27) cause a non-ulcerative infection in BALB/c mice with a pattern of mild swelling which progresses slowly, followed usually by regression [13, 38–40]. It should also be noted that we used un-selected stationary phase promastigotes instead of metacyclic promastigotes to challenge the mice. Since the percentage of the metacyclic parasite in the stationary phase was not high (~25–35%), low pathogenicity, especially in terms of lesion diameter may have been caused.

No parasite was seen in any spleen samples of our experimental groups. This result is consistent with previous reports in which the parasite was absent in visceral organs (spleen or liver) of BALB/c mice after infection by *L*. *tropica* strain KK27 [13]. However, in contrast to these results, we had previously observed the visceralization of the same strain into spleen of BALB/c mice [11, 39]. Thus, it appears that the dissemination of *L*. *tropica* KK27 to the visceral organs is an arguable issue and more studies are needed to clarify it.

The parasite load results regarding the mice lymph nodes clearly show that MPL was a potent adjuvant which decreased the parasite load when used beside SLA or recombinant LtSTI1. In contrast, R848 had no effect on ability of LtSTI1 to reduce the parasite load and it even increased the parasite load when used alongside SLA. Although there is no other report about the effects of MPL and R848 on protection by *L*. *tropica* antigens in BALB/c mice, the present findings were similar to our previous work on *L*. *major* in which we have shown and discussed that MPL is a better adjuvant in comparison to R848 when used with *L*. *major* SLA [20]. Our findings also suggest that recombinant LtSTI1 was a better vaccine candidate than SLA. As we are aware, there is no report about vaccine efficacy of *L*. *tropica* SLA and LtSTI1 and this is the first report that indicates a recombinant protein of *L*. *tropica* confers a better protection as a vaccine candidate than *L*. *tropica* whole-cell antigens.

The cytokine assays show that the levels of the cytokines responses in the experimental groups before the challenge and at week-8 post challenge were very low. At week-16 post challenge, the cytokines levels of our experimental groups were significantly higher than the negative controls (un-stimulated lymphocytes). The only clear result in this regard is that LtSTI1 is more able to evoke cytokine responses in comparison to SLA. There is only one well-documented report on mice cytokine responses against *L*. *tropica* strain KK27, provided by Anderson et al. [40], in which due to very weak immune responses of this strain, antigen-loaded bone marrow derived dendritic cells have been applied in order to increase the in vitro cytokine level. Also, it is possible that the setting of our in vitro cytokine assessment was not optimized to detect the low cytokine responses.

Since both the cytokines responses and the lymph node parasite load results of our experimental groups were higher at 16-week post challenge (in comparison to 8-week post challenge assay), it could be concluded that the immune responses of BALB/c mice toward *L*. *tropica* strain KK27 infection are delayed and develop slowly, similar to the delayed pattern of the parasite’s pathogenesis. Similar to these findings, the delayed pathogenesis of *L*. *tropica* strain KK27 in comparison to *L*. *major*, has been previously reported by Lira et al. [13]. They have shown that the pathogenesis of *L*. *tropica* strain KK27 in BALB/c mice can be observed approximately 120 days after challenging the mice with 1 million stationary promastigotes; however, they did not determine the cytokines responses.

A low level of the immune responses, detected by DTH assay (without cytokine measurements) was also seen in our previous work [41]. There, we observed that DTH response induced in BALB/c mice by *L*. *tropica* was much weaker in comparison to *L*. *major*. Considering that DTH is a cell- and cytokine-mediated immune response [36, 42], it could be concluded that *L*. *tropica* cause a mild immune response in BALB/c mice.

The level of secreted IL-10 by group LR at week-16 post challenge was slightly higher than the levels quantified for LM group. As previously shown, IL-10 is able to deactivate IFN-γ-mediated killing by the macrophages, leading to suppression of Th1 responses which is critical for fighting against leishmaniases [43, 44]. Thus, it seems that effective adjuvants should be able to reduce the secretion of IL-10 by the host. There are not enough data on cytokine responses against *L*. *tropica*; however, it has been clearly shown that the immune responses against *L*. *major* infection is controlled by IL-10 [45, 46]. Thus, it seems that R848 is not an effective adjuvant due to its induction of IL-10 production. Considering the low immune responses generated against *L*. *tropica* in the present study (as discussed above), it appears that further investigations are necessary to clarify this issue.

Since the short-term results showed that recombinant LtSTI1 is a better vaccine candidate in comparison to SLA, we focused on this promising antigen and assayed the long-term immune responses raised by it. Our long-term assay on the parasite load showed that none of our vaccines were strong enough to decrease the parasite load in comparison to PBS group, although LM group had a significantly lower parasite load than LR group. Altogether, the long-term assay suggests that although MPL was a more potent adjuvant than R848 for LtSTI1, its effectiveness on mice didn’t last for up to 16 weeks after the last boost. Due to their low amounts, the cytokine responses after the challenge within the long-term period were not conclusive, similar to the results obtained during the short-term assay (*i*.*e*. 8 weeks after the challenge). Moreover, considering the reports showing that protection assessed by needle and sand fly challenges are not necessary the same [47, 48] and the fact that we used needle challenge in our study, the effectiveness of our vaccination strategies against sand fly challenge needs further study.

## Conclusion

To our best knowledge, this is the first report on immunization formulations *against L*. *tropica*, composed of the parasite’s whole-cell killed antigens (SLA) or its recombinant protein (LtSTI1) antigen, used with MPL and R848 adjuvants. We successfully prepared the SLA and also isolated, cloned, expressed and purified LtSTI1 and applied their combinations as vaccine candidates against *L*. *tropica* infection in a BALB/c mice model of infection. Our data indicate that MPL, in contrast to R848, was more effective with the antigens to evoke immune responses and it specifically worked better with LtSTI1 to evoke DTH.

Altogether, our data suggest that LtSTI1 and MPL make a promising antigen/adjuvant combination for further studies on vaccination strategies against *L*. *tropica*. Furthermore, the data of the present study, as first steps on a vaccination strategy against *L*. *tropica*, will broaden insights on pathology and immunology of this neglected pathogen.
